# Nation related participation and performance trends in ‘Ironman Hawaii’ from 1985 to 2012

**DOI:** 10.1186/2052-1847-6-16

**Published:** 2014-04-15

**Authors:** Philippe Dähler, Christoph Alexander Rüst, Thomas Rosemann, Romuald Lepers, Beat Knechtle

**Affiliations:** 1 Institute of General Practice and for Health Services Research, University of Zurich, Zurich, Switzerland; 2 INSERM U1093, Faculty of Sport Sciences, University of Burgundy, Dijon, France; 3 Gesundheitszentrum St. Gallen, Vadianstrasse 26, 9001 St. Gallen, Switzerland

**Keywords:** Triathlon, Nationality, Finisher, Swimming, Cycling, Running

## Abstract

**Background:**

This study examined participation and performance trends in ‘Ironman Hawaii’ regarding the nationality of the finishers.

**Methods:**

Associations between nationalities and race times of 39,706 finishers originating from 124 countries in the ‘Ironman Hawaii’ from 1985 to 2012 were analyzed using single and multi-level regression analysis.

**Results:**

Most of the finishers originated from the United States of America (47.5%) followed by athletes from Germany (11.7%), Japan (7.9%), Australia (6.7%), Canada (5.2%), Switzerland (2.9%), France (2.3%), Great Britain (2.0%), New Zealand (1.9%), and Austria (1.5%). German women showed the fastest increase in finishers (r^2^ = 0.83, p < 0.0001), followed by Australia (r^2^ = 0.78, p < 0.0001), Canada (r^2^ = 0.78, p < 0.0001) and the USA (r^2^ = 0.69, p < 0.0001). Japanese women showed no change in the number of finishers (r^2^ = 0.01, p > 0.05). For men, athletes from France showed the steepest increase (r^2^ = 0.85, p < 0.0001), followed by Austria (r^2^ = 0.68, p < 0.0001), Australia (r^2^ = 0.67, p < 0.0001), Brazil (r^2^ = 0.60, p < 0.0001), Great Britain (r^2^ = 0.46, p < 0.0001), Germany (r^2^ = 0.26, p < 0.0001), the United States of America (r^2^ = 0.21, p = 0.013) and Switzerland (r^2^ = 0.14, p = 0.0044). The number of Japanese men decreased (r^2^ = 0.35, p = 0.0009). The number of men from Canada (r^2^ = 0.02, p > 0.05) and New Zealand (r^2^ = 0.02, p > 0.05) remained unchanged. Regarding female performance, the largest improvements were achieved by Japanese women (17.3%). The fastest race times in 2012 were achieved by US-American women. Women from Japan, Canada, Germany, Australia, and the United States of America improved race times. For men, the largest improvements were achieved by athletes originating from Brazil (20.9%) whereas the fastest race times in 2012 were achieved by athletes from Germany. Race times for athletes originating from Brazil, Austria, Great Britain, Switzerland, Germany, Australia, Canada, Japan, New Zealand and France decreased. Race times in athletes originating from Australia and the United States of America showed no significant changes. Regarding the fastest race times ever, the fastest women originated from the United States (546 ± 7 min) followed by Great Britain (555 ± 15 min) and Switzerland (558 ± 8 min). In men, the fastest finishers originated from the United States (494 ± 7 min), Germany (496 ± 6 min) and Australia (497 ± 5 min).

**Conclusions:**

The ‘Ironman Hawaii’ has been dominated by women and men from the United States of America in participation and performance.

## Background

Triathlon is a multi-sport combining the three disciplines swimming, cycling and running [1,2]. Among the various distances established nowadays, the Ironman triathlon such as the ‘Ironman Hawaii’ consists of 3.8 km swimming, 180.2 km cycling and 42.2 km running [3] and counts as one of the top tier endurance races worldwide [4]. The first Ironman triathlon was held in Hawaii in 1978 and has evolved to become the ‘Ironman Triathlon World Championship’ held annually in October [5]. There are limited slots for elite and non-elite triathletes and potential participants for ‘Ironman Hawaii’ have to qualify in one of currently 30 official qualifier events around the world [6-8]. Since the beginning of the ‘Ironman Hawaii’ in 1978, participation has increased and today more than 1,800 athletes compete annually in this race [5].

In recent years, even longer races called ultra-triathlons have been established [9]. With the growing popularity of long-distance races, extensive research in various aspects potentially influencing performance has already been conducted. For example, the effects of gender [10,11], age [12-14], anthropometry [15-17], training [18] and previous experience [19-21] on ‘Ironman’ performance have been examined.

Apart from these aspects, the nationality of the participants might of importance as it has been shown for ultra-marathon running [22]. In multi-sports athletes such as triathletes, several studies assessed the aspects of nationality in long-distance triathlons such as ‘Ironman’ and longer distances [9,22-26]. For example, Jürgens et al. [24] showed that ‘Ironman Switzerland’ as one of the European qualifying races for the ‘Ironman Hawaii’ has been dominated by central European triathletes regarding both participation and performance. Rüst et al. [26] recently showed that European athletes dominated Double Iron ultra-triathlons covering 7.6 km swimming, 360 km cycling and 84.4 km running although US-Americans were to first to hold this kind of races. Lenherr et al. [9] reported that European athletes accounted for ~80% of the participants in all Double Iron to Double Deca Iron ultra-triathlons held from 1985 to 2011 and provided the largest number of winners as well. Therefore, it seems that European triathletes dominate long-distance triathlons since most of these races were held in Europe [9,23,25,26].

While it is suggested that American triathletes dominated the ‘Ironman Hawaii’ in the beginning [27], the further participation and performance trends are unknown. The aim of this study was to examine the changes in participation and overall race times in the ‘Ironman Hawaii’ regarding the nationality of the athletes from 1985 to 2012. We hypothesized that triathletes from the United States of America would dominate this race regarding participation and performance but that Europeans would reduce the gap in participation and total performance over the years.

## Methods

### Ethics

All procedures used in the study were approved by the Institutional Review Board of Kanton St. Gallen, Switzerland with a waiver of the requirement for informed consent of the participants given the fact that the study involved the analysis of publicly available data.

### Data collection and analysis

Data from all finishers in ‘Ironman Hawaii’ between 1978 and 2012 were obtained from the race website [5]. Since for the first seven years of the event no data about nationality were available in the rankings, only data from 1985 and later could be considered for the analysis. Overall race time and nationality were available from 39,695 finishers originating from 124 countries, including 8,973 women and 30,722 men. Since not all rankings reported non-finishers in all years, non-finishers were not considered for analyses. From 11 men, no information about nationality was given and thus had to be excluded from analysis. All athletes originating from countries with at least ten annual female and male finishers in at least 20 of the 28 years were further analysed regarding the changes in the number of finishers and performance trends. For quality purposes, athletes from countries providing less than ten female or male finishers in more than one third during the 1985-2012 period were excluded from this analysis. For women, athletes from Australia, Canada, Germany, Japan and the United States of America (*i.e.* 85.1% of all female finishers) could be included. For men, athletes from Australia, Austria, Brazil, Canada, France, Great Britain, Germany, Japan, New Zealand, Suisse and the United States of America (*i.e.* 89.8% of all male finishers) were included. From each of these countries, the annual ten fastest women and men were analysed regarding their overall race times and the changes in overall race times across years. When less than ten finishers could be analysed per country, year and gender, the respective year was excluded from analysis. From the 23 countries providing a total (*i.e.* all men and all women) of more than 100 finishers (*i.e.* representing 97.8% of all female and 96.8% of all male finishers), the overall ten best female and male finishers were determined and analysed regarding differences in performance.

### Statistical Analysis

In order to increase the reliability of data analysis, each set of data was tested for normal distribution as well as for homogeneity of variances in advance of statistical analysis. Normal distribution was tested using a D’Agostino and Pearson omnibus normality test and homogeneity of variances was tested using a Levene’s test. Linear regression analyses were used to find significant changes in the change of a variable across years. Single and multi-level regression analyses were used to investigate changes in performance. A hierarchical regression model was used to avoid the impact of a cluster-effect on results in case one athlete finished more than once in the annual top ten. A one-way analysis of variance (ANOVA) with subsequent Tukey-Kramer post-hoc analysis was applied to find differences between multiple groups (*i.e.* comparison of race times between different countries). Statistical analyses were performed using IBM SPSS Statistics (Version 22, IBM SPSS, Chicago, IL, USA) and GraphPad Prism (Version 6.01, GraphPad Software, La Jolla, CA, USA). Significance was accepted at p < 0.05 (two-sided for *t*-tests). Data in the text are given as mean ± standard deviation (SD).

## Results

### Participation trends

**Figure 1:**
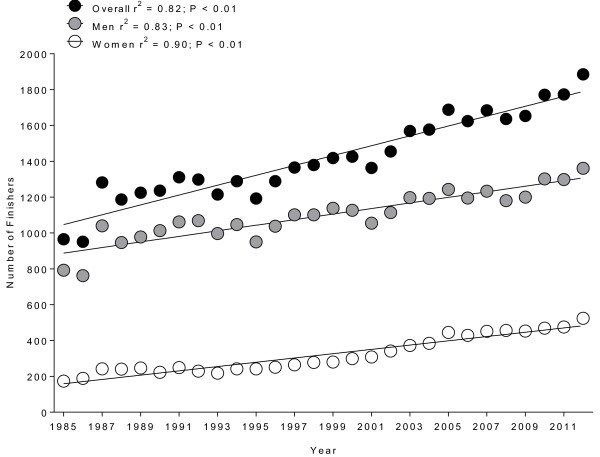
Change in the annual number of men, women and overall finishers ‘Ironman Hawaii’ from 1985 to 2012.

A total of 39,706 triathletes finished ‘Ironman Hawaii’ between 1985 and 2012 including 8,973 women (22.6%) and 30,733 men (77.4%). During the studied period, a total of 12,904 triathletes with 2,611 women (20%) and 10,293 men (80%) finished the race more than once. During these 27 years, the overall number of finishers increased from 965 (1985) to 1,885 (2012). The number in women increased from 173 (1985) to 524 (2012) and in men from 792 (1985) to 1,361 (2012) (Figure 1).

**Figure 2:**
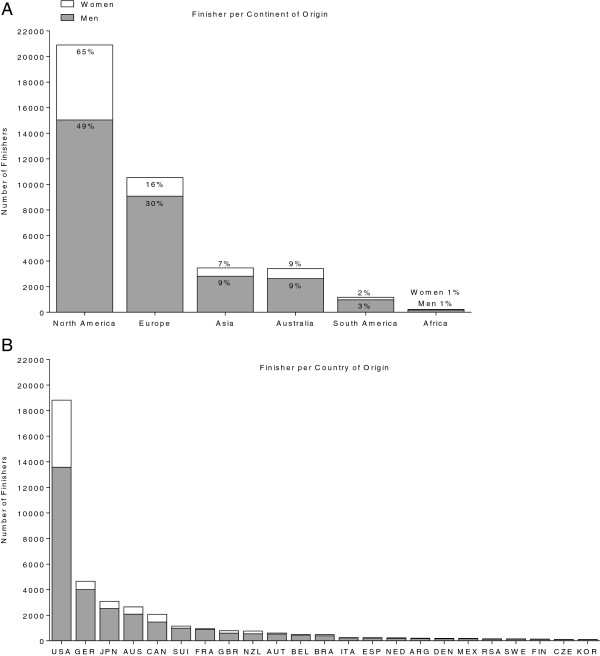
**Number of female and male finishers per continent of origin in ‘Ironman Hawaii’ (Panel A) and number of female and male finishers per country of origin (Panel B).** USA = United States of America, GER = Germany, JPN = Japan, AUS = Australia, CAN = Canada, SUI = Switzerland, FRA = France, GBR = Great Britain, NZL = New Zealand, AUT = Austria, BEL = Belgium, BRA = Brazil, ITA = Italy, ESP = Spain, NED = Netherlands, ARG = Argentina, DEN = Denmark, MEX = Mexico, RSA = Republic South Africa, SWE = Sweden, Fin = Finland, CZE = Czech Republic, KOR = Korea.

Figure 2 presents the numbers of finishers regarding their continent (Panel A) and country (Panel B) of origin. Among all female and male finishers, the majority (52.6%) of successful finishers originated from North America (Figure 2A). A total of 26.5% of athletes originated from Europe, 8.7% from Asia, 8.6% from Australia, 2.9% from South America and 0.6% from Africa. Regarding the genders, the dominance of North American women with 65% of all finishers is even more obvious. In men, 49% of all finishers originated from North America. The second largest group of European triathletes provided 30% male and 16% female triathletes.

Regarding the countries of origin of the athletes (Figure 2B), the ten countries with the highest numbers of overall finishers were the United States of America (18,824; 47.5%), followed by Germany (4,664; 11.7%), Japan (3,089; 7.9%), Australia (2,649; 6.7%), Canada (2,077; 5.2%), Switzerland (1,152; 2.9%), France (942; 2.3%), Great Britain (784; 2.0%), New Zealand (762; 1.9%) and Austria (610; 1.5%). Triathletes from North America originated mainly from the United States of America (90%), while the largest European group originated from Germany (45%). All other continents also showed the dominance of one nation; in Africa triathletes of the Republic of South Africa (80%), in Asia athletes from Japan (90%), in Oceania athletes from Australia (77%) and in South America athletes from Brazil (41%).

In men, finishers from four continents (*i.e.* Europe, Australia, South America and Africa) showed a significant increase in the number of triathletes from 1985 to 2012. This was not the case for North American triathletes. In women, triathletes from five continents (*i.e.* Europe, North America, Australia, South America and Africa) showed an increase in the number of finishers. North American women represented the largest group of finishers (5,550; 66%) followed by Europeans (1,350; 16%). For both women and men, European athletes showed the steepest increase. Participation from African and Asian triathletes remained unchanged. Nonetheless, North American triathletes still represented the largest group in 2012, leaving European triathletes in second place.

**Figure 3:**
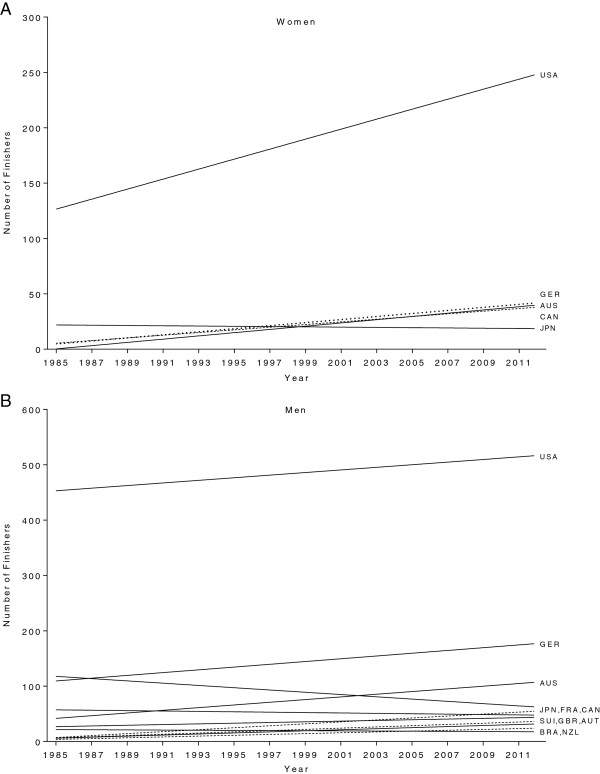
Changes in the annual numbers of female (Panel A) and male (Panel B) finishers by origin.

Figure 3 shows the increase in the numbers of finishers across time for women (Panel A) and men (Panel B) regarding their country of origin. German women showed the fastest increase in finishers from 0 (1985) to 31 (2012) (r^2^ = 0.83, p < 0.0001), followed by athletes from Australia with 3 (1985) to 62 (2012) (r^2^ = 0.78, p < 0.0001), Canada with 11 (1985) to 38 (2012) (r^2^ = 0.78, p < 0.0001) and the United States of America with 146 (1985) to 269 (2012) (r^2^ = 0.69, p < 0.0001) (Figure 3A). Japanese women showed no change in the number of finishers across years (r^2^ = 0.01, p > 0.05) with a mean of 20 annual finishers.

For men (Figure 3B), athletes from France showed the steepest increase from 1 (1985) to 46 (2012) finishers (r^2^ = 0.85, p < 0.0001), followed by athletes from Austria with 2 (1985) to 24 (2012) finishers (r^2^ = 0.68, p < 0.0001), Australia with 33 (1985) to 164 (2012) finishers (r^2^ = 0.67, p < 0.0001), Brazil with 4 (1985) to 36 (2012) finishers (r^2^ = 0.60, p < 0.0001), Great Britain with 17 (1985) to 52 (2012) finishers (r^2^ = 0.46, p < 0.0001), Germany with 26 (1985) to 116 (2012) finishers (r^2^ = 0.26, p < 0.0001), the United States of America with 511 (1985) to 523 finishers (2012) (r^2^ = 0.21, p = 0.013) and Switzerland from 7 (1985) to 39 (2012) finishers (r^2^ = 0.14, p = 0.0044). The number of Japanese men decreased from 73 (1985) to 33 (2012) finishers (r^2^ = 0.35, p = 0.0009). The number of Canadian men remained unchanged at 53 finishers (r^2^ = 0.02, p > 0.05) and the number of male athletes originating from New Zealand was unchanged at 20 finishers (r^2^ = 0.02, p > 0.05).

### Performance trends

**Figure 4:**
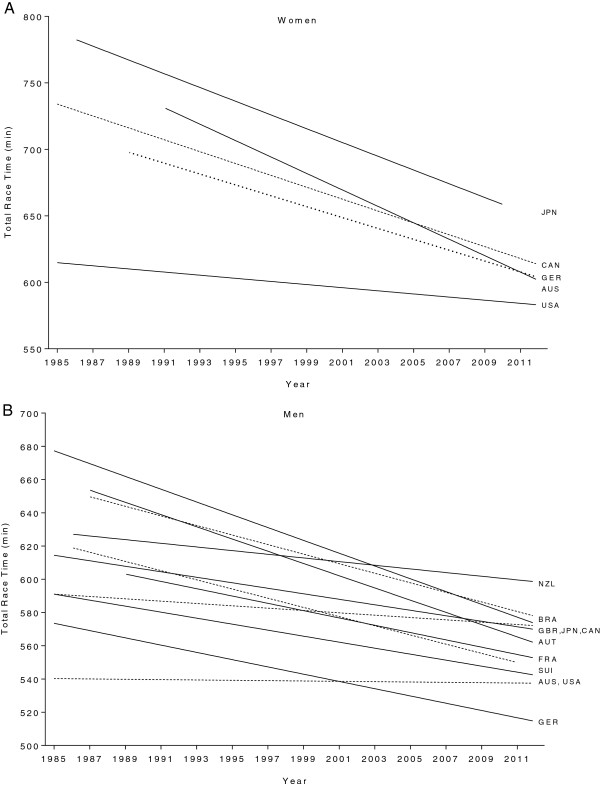
Changes in race times of the annual ten fastest female (Panel A) and male (Panel B) finishers by origin.

Figure 4 presents the changes in race times for the annual ten fastest women (Panel A) and men (Panel B). In women, the largest improvements were achieved by Japanese women (Figure 4A) with an improvement by 17.3% between 1985 and 2010 (Table 1). The fastest race times in 2012 were achieved, however, by US-American women with 578 ± 14 min (Table 1A). Women from Japan, Canada, Germany, Australia, and the United States of America improved their race times between 1985 and 2012 (Figure 4A) also when controlled for multiple finishes within the top ten (Table 1B and 1C).

**Table 1:** Changes in race times for women

**A: Change in race times for the annual top ten women between 1985 and 2012**					
**Country**	**Race time in 1985 (min)**	**Race time in 2012 (min)**	**Change absolute (min)**	**Change in percent (%)**	
Japan^#^	869 ± 97	718 ± 124	−150 ± 101	−17.3	
Canada	778 ± 75	651 ± 32	−127 ± 51	−16.3	
Germany^*^	712 ± 68	613 ± 28	−99 ± 42	−13.9	
Australia^#^	709 ± 71	619 ± 21	−89 ± 52	−12.5	
United States	651 ± 16	578 ± 14	−73 ± 18	−11.2	
**B: Multi-level regression analyses for changes in race times for the annual top ten women between 1985 and 2012**					
**Country**	***ß***	**SE ( *ß *)**	**Stand. *ß***	**T**	**P**
Japan^#^	−5.152	0.528	−0.527	−9.763	< 0.001
Canada	−4.932	0.521	−0.523	−9.472	< 0.001
Germany^*^	−4.310	0.422	−0.560	−10.205	< 0.001
Australia^#^	−6.441	0.642	−0.591	−10.034	< 0.001
United States	−1.180	0.192	−0.352	−6.149	< 0.001
**C: Multi-level regression analyses for change in race times for the annual top ten women between 1985 and 2012 with correction for multiple participations**					
**Country**	***ß***	**SE ( *ß *)**	**Stand. *ß***	**T**	**P**
Japan^#^	−5.152	0.528	−0.527	−9.763	< 0.001
Canada	−4.932	0.521	−0.523	−9.472	< 0.001
Germany^*^	−4.310	0.422	−0.560	−10.205	< 0.001
Australia^#^	−6.441	0.642	−0.591	−10.034	< 0.001
United States	−1.180	0.192	−0.352	−6.149	< 0.001

^*^ = For German women, the first year with at least ten finishers was in 1989, ^#^ = for Australian women, the first year with at least ten finishers was in 1991, ^#^ = for Japanese women, 2010 was the last year with at least ten finishers.

For men (Figure 4B), the largest improvements were achieved by athletes originating from Brazil (Table 2A) with 20.9% faster race times in 2012 compared to 1985 whereas the fastest race times in 2012 were achieved by athletes from Germany (Table 2A). Race times for athletes originating from Brazil, Austria, Great Britain, Switzerland, Germany, Canada, Japan, New Zealand and France decreased between 1985 and 2012 (Figure 4B) also when corrected for multiple finishes in the top ten (Table 2B and 2C). Race times in athletes originating from Australia and the United States of America showed, however, no significant changes across years (567 ± 22 and 541 ± 18 min, respectively).

**Table 2:** Changes in race times for men

**A: Change in race times for the annual top ten men between 1985 and 2012**					
**Country**	**Race time in 1985 (min)**	**Race time in 2012 (min)**	**Change absolute (min)**	**Change in percent (%)**	
Brazil^#^	741 ± 118	586 ± 14	−155 ± 31	−20.9	
Austria^*^	693 ± 76	579 ± 11	−114 ± 24	−16.4	
Great Britain	695 ± 21	586 ± 23	−109 ± 24	−15.7	
Switzerland^#^	652 ± 39	549 ± 21	−103 ± 23	−15.5	
Germany	627 ± 34	537 ± 24	−89 ± 15	−14.2	
Australia	629 ± 19	540 ± 22	−89 ± 22	−14.1	
Canada	639 ± 20	583 ± 27	−56 ± 21	−8.7	
United States	576 ± 19	538 ± 17	−39 ± 18	−6.7	
Japan	651 ± 19	617 ± 16	−34 ± 17	−5.2	
New Zealand	652 ± 39	630 ± 44	−22 ± 39	−3.3	
France^§^	569 ± 33	551 ± 23	−18 ± 22	−3.1	
**B: Multi-level regression analyses for changes in race times for the annual top men between 1985 and 2012**					
**Country**	***ß***	**SE ( *ß *)**	**Stand. *ß***	**T**	**P**
Brazil^#^	−4.183	0.412	−0.472	−10.123	< 0.001
Austria^*^	−3.872	0.387	−0.561	−9.999	< 0.001
Great Britain	−4.012	0.328	−0.646	−12.221	< 0.001
Switzerland^#^	−2.760	0.251	−0.565	−11.012	< 0.001
Germany	−2.360	0.203	−0.580	−11.647	< 0.001
Canada	−1.765	0.244	−0.404	−7.237	< 0.001
Japan	−1.847	0.231	−0.439	−7.985	< 0.001
New Zealand	−1.847	0.231	−0.439	−7.985	< 0.001
France^§^	−2.209	0.327	−0.408	−6.747	< 0.001
**C: Multi-level regression analyses for change in race times for the annual top men between 1985 and 2012 with correction for multiple participations**					
**Country**	***ß***	**SE ( *ß *)**	**Stand. *ß***	**T**	**P**
Brazil^#^	−4.183	0.412	−0.472	−10.123	< 0.001
Austria^*^	−3.872	0.387	−0.561	−9.999	< 0.001
Great Britain	−4.012	0.328	−0.646	−12.221	< 0.001
Switzerland^#^	−2.760	0.251	−0.565	−11.012	< 0.001
Germany	−2.360	0.203	−0.580	−11.647	< 0.001
Canada	−1.765	0.244	−0.404	−7.237	< 0.001
Japan	−1.847	0.231	−0.439	−7.985	< 0.001
New Zealand	−1.847	0.231	−0.439	−7.985	< 0.001
France^§^	−2.209	0.327	−0.408	−6.747	< 0.001

^#^= for Swiss men, the first year with at least ten finishers was in 1988, ^*^= for Austrian men, the first year with at least ten finishers was in 1987, ^§^= for French men, the first year with ten finishers was in 1989, ^#^= for Brazilian men, the first year of ten finishers was in 1987, ^¶^= for athletes from New Zealand, the first year with ten men was 1986.

**Figure 5:**
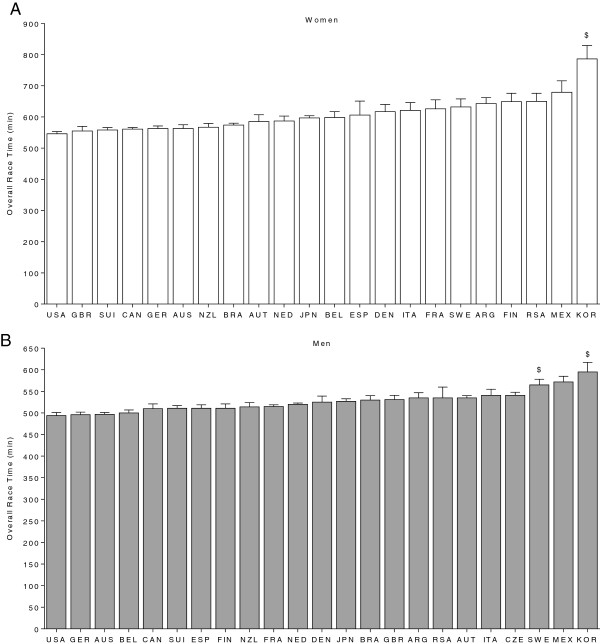
**Overall race time of top ten finishers per country for women (Panel A) and men (Panel B).** $ = significantly slower than next faster country. USA = United States of America, GER = Germany, JPN = Japan, AUS = Australia, CAN = Canada, SUI = Switzerland, FRA = France, GBR = Great Britain, NZL = New Zealand, AUT = Austria, BEL = Belgium, BRA = Brazil, ITA = Italy, ESP = Spain, NED = Netherlands, ARG = Argentina, DEN = Denmark, MEX = Mexico, RSA = Republic South Africa, SWE = Sweden, Fin = Finland, CZE = Czech Republic, KOR = Korea.

In Figure 5, the fastest race times achieved between 1985 and 2012 sorted by nations are presented for women (Panel A) and men (Panel B). In women, finishers from the United States of America (546 ± 7 min) were fastest followed by finishers from Great Britain (555 ± 15 min) and Switzerland (558 ± 8 min). In men, the fastest finishers originated from the United States of America (494 ± 7 min), Germany (496 ± 6 min) and Australia (497 ± 5 min).

## Discussion

This study intended to examine the changes in participation and race times in ‘Ironman Hawaii’ from 1985 to 2012 regarding the nationality of the athletes. It was hypothesized that triathletes from the United States of America would dominate ‘Ironman Hawaii’ regarding participation and performance but that Europeans would reduce the gap in participation and performance over the years. The most important findings were that (*i*) most of the finishers originated from the United States of America, (*ii*) the number of finishers increased to a higher extent in athletes from European countries and Australia than from the United States of America and (*iii*) the fastest race times were achieved by female and male finishers originating from the United States of America.

### Half of all finishers originated from the United States of America

A first important finding was that according to our hypothesis the majority of finishers originated from North America and more precisely from the United States of America for both men and women. However, over the 27-year period, participation in female and male European athletes grew significantly. A possible explanation for the North American dominance could be the number of qualifying events in each region. As Table 3 shows, in 2012 most qualifying races were held in North America; 16 in totals with 777 Ironman World Championship slots to give away. Europe came in second place offering 465 slots out of 7 races. All other continents offered far fewer choices to gather a slot.

**Table 3:** Qualifying events in 2012 for ‘Ironman Hawaii’

**Continent**	**Ironman Hawaii slots**	**Number of races**
North America	777	16
Europe	465	7
Australia/Oceania	250	5
Asia	120	3
South America	100	2
Africa	30	1
**Total**	**1’742**	**30**

Differences regarding participation and performance for athletes competing in ‘Ironman Hawaii’ and in the qualifier races have already been investigated [7,8]. Considering the nationality of Ironman triathletes intending to qualify for ‘Ironman Hawaii’, US-American triathletes dominated both participation and performance in both ‘Ironman Hawaii’ and its qualifiers [7]. Differences regarding participation and performance trends in different age groups between ‘Ironman Hawaii’ and its qualifier races have already been described [8]. Triathletes aged 25-49 years and men were generally underrepresented in ‘Ironman Hawaii’ compared to the Ironman qualifier races. These athletes may have had less chance to qualify for ‘Ironman Hawaii’ than female ‘ or younger (<25 years) and older (>50 years) athletes [8]. These differences are most probably due to the higher number of qualifying slots in the United States of America compared to other countries.

The population of the different countries might also be a reason why more US-American athletes finish in ‘Ironman Hawaii’ compared to athletes from other countries. We therefore investigated a potential correlation between population size in 2012 and number of total finishers from 1985 and 2012 (Table 4) and were able to show a significant correlation between population size and number of total finishers in men (r = 0.91, p = 0.0002) and women (r = 0.89, p = 0.0006). Therefore, a triathlete originating from a larger country has a better chance to start and finish in ‘Ironman Hawaii’, which could be another reason for the dominance of US-American triathletes.

**Table 4:** The number of total finishers in ‘Ironman Hawaii’ 1985-2012 and in percent of total population

**Country**	**Population in 2012**^**1**^	**Total finishers overall**	**Finishers in 2012**	**Total finishers in percent of population in 2012**	**Finishers in 2012 in percent of population in 2012**
United States	317,546,000	18,824	792	0.0059	0.00024
Germany	80,586,000	4,664	147	0.0057	0.00018
Japan	127,220,000	3,089	40	0.0024	3.1442 e^−05^
Australia	23,383,930	2,649	226	0.011	0.00096
Canada	35,295,770	2,077	111	0.0058	0.00031
Switzerland	8,112,200	1,152	58	0.014	0.00071
France	65,820,916	942	51	0.0014	7.7483 e^−05^
Great Britain	63,705,000	787	76	0.0012	0.00011
New Zealand	4,513,090	762	31	0.0168	0.00068
Austria	8,501,502	610	32	0.0071	0.00037

^1^OECD, Organisation for Economic Cooperation and Development. http://www.oecd.org[28].

An interesting finding was that the number of athletes from European countries, Australia and Brazil showed the highest increase across years. One of the reasons for the increasing European participation could be an especially high interest in Ironman triathlon. Central Europe is now the region with the highest concentration of qualifying races [6-8]. In addition, Jürgens et al. [24] showed that two thirds of the participants in a European qualifier such as the ‘Ironman Switzerland’ originated from Switzerland and its neighboring countries. The fact that Switzerland is in the Centre of Europe might explain this. Another possible sign of the broad interest in Ironman triathlon across Europe is the fact that according to our data a considerable number of finishers of the ‘Ironman Hawaii’ originated from several central European countries besides Germans as the largest European group, such as Switzerland, France, and Austria.

Another reason for the increase in European participation could be the fact that mainly Europeans were able to improve their performance and therefore gathered more slots. It has to be kept in mind that the finishers in ‘Ironman Hawaii’ come from a highly pre-selected field where obtaining a slot at a qualification race leads to participation [7,8]. Slots are given away not based on an absolute time limit since with the given topographical and climatic differences of each race course they are not comparable. In contrast, slots are given away based on the individual race ranking. Each of these qualifying races has a different, predefined amount of slots that can be won.

Our data further showed that overall and both male and female participation grew significantly over time. This is in accordance with the well-established finding that interest in endurance sports has grown considerably worldwide [29,30]. One could argue that the participation in ‘Ironman Hawaii’ is limited by the number of slots available and a generalization of this finding is not legitimate. However, not only has the number of triathletes at qualifying races all over the world risen constantly but so has the number of these races as well.

### Participation trends differ between countries and continents

The influence of nationality and ethnic background on performance is best known from East-African distance runners. Kenyans have been dominating middle and long-distance races, especially the world cross-country championship, for a long time [31]. Ethiopian athletes boast a success record in international distance running second only to Kenya [32]. Wilber and Pitsiladis [33] proposed a multifactorial model taking genetic, physiological and psychological factors into account. For these athletes, a successful running career gives them a unique opportunity to leave poverty behind and reach the highest socioeconomic level in their societies. It was shown previously that for Kenyan runners economic success was the most important motivation [34].

However, participation from triathletes originating from Australia and especially South America and Africa in men and women grew only slightly. Triathletes from Asia were barely able to keep the number of participants over time. For Japanese triathletes, for example, the annual number of female finishers remained unchanged whereas the number of male finishers decreased. Socioeconomic factors could be one of the reasons for this finding. The limited data that exists on demographic factors in long-distance athletes shows them to have a higher than average education and income than the average individual [35,36]. This could on the one hand well be a precondition to participate in this sport. With Ironman races being held all over the world and the ‘World Championship’ in Hawaii there has to be sufficient funds for travelling and time spent abroad. And in order to achieve a performance level sufficient to qualify for the World Championship race, long training intervals in all three disciplines are necessary [15,16,20]. It is therefore probable that non-full-time professional athletes have to cut back their work hours and as a consequence earn less. On the other hand it is questionable if the stagnation of participation from many regions can be explained with socioeconomic factors alone.

Participants from Asia were mainly Japanese triathletes (*i.e.* 90% of all) with an additional 4% coming from Hong Kong and Singapore, all industrialized high-income countries [37]. The same is true for African finishers where South Africans dominate with 80% and obviously for Australian triathletes. In South American triathletes Brazil dominates with 41%, Argentina and Mexico coming in second place with 17% each. These three countries are classified as upper middle income countries by the World Bank [37]. Therefore, one could also argue that the limited potential pool of less developed regions has already been exhausted as for most triathletes from these countries participation in Ironman triathlon is too expensive. Another factor could be a possible lack of training facilities, especially for swimming and cycling. In addition weather and climatic factors could play a role, since cycling and running mainly happens outdoors. Topographical factors could be an issue as well, for example lack of uphill grades.

### Participation trends in women

Female participation grew slightly faster than men, but men still dominate largely. Sex differences in competitiveness have already been assessed in long-distance races [38]. Sociocultural as well as evolutionary factors have been described. One explanation of our data could be the fact that with changing roles of women in society, *e.g.* higher education and participation in the workforce, there has also been a convergence of competitiveness of men and women in long-distance sports. The still remaining considerable gap in participation could be attributed to evolutionary differences in the sense that men still have a greater training motivation than women [38].

Another outcome of the selection process is that there is a higher share of women competing in the ‘Ironman Hawaii’ than in any other Ironman race. In 2012, 36% of all finishers were women in the ‘Ironman Hawaii’ while for example in ‘Ironman Switzerland’, Zurich, women counted for only 14% or 22% in St. George, UT, USA [5]. Compared to US-American men, female participation grew considerably, as was true for European women. They therefore count for a considerable share of the total participation growth. This hints to an increasing interest in long-distance triathlons in women in these regions. Historically, women have been largely underrepresented and this trend could be seen as a move to correct this situation.

### US-American dominance in performance

As we anticipated, triathletes originating from the United States of America dominated the race from the beginning and achieved the fastest race times. Results showed no change after correction for multiple participation of the same athlete. This comes as no surprise since this discipline was invented in the United States of America. This correlation can be seen in other long-distance disciplines, for example in ultra-triathlons, where US-Americans started the races but Europeans dominated [25,26]. This might be explained with an advantage in experience, an already established structure of professional sport associations and existing ‘role models’ for aspiring future competitors. However, between 1985 and 2012, male triathletes originating from the United States of America were not able to improve their performance.

In women, the top ten triathletes from the United States of America were able to improve their overall race time significantly and to achieve the fastest race times over the whole period. Female triathletes of all five analyzed nations showed a significant improvement. Results showed no change after correction for multiple participation of the same athlete. An explanation could be the fact that the ‘Ironman Triathlon’ traditionally was not an established discipline in women sports and the performance level is still advancing due to growing interest.

Another interesting finding was that the top ten German male triathletes showed the second fastest race times. German triathletes were able to improve their overall race times to a higher extent compared to triathletes from the United States of America. This finding is well in line with the European increase in participation and likely a sign of high interest in this sport. In Europe, the first race was held 1992 in Lanzarote, Spain [39]. Long before, the European Championship in triathlon was held in Roth, Germany [40]. The performance of German triathletes in the ‘Ironman Hawaii’ might therefore be explained with this long tradition in triathlon. Another factor could also be the population size. Germany has the largest population in Western Europe [41] and therefore the talent pool is larger than in other European countries. For smaller nations it is more challenging to provide the ten top triathletes that were analyzed. Additionally, the fact that German triathletes are still improving shows that no physiological limit has been reached yet.

### Limitations

Since only those nations providing more than ten finishers per year per gender could be considered for further analysis and although this lead to the inclusion of most triathletes, many countries had to be excluded. It is therefore possible that a nation provides a small but excellent group of triathletes that could not have been assessed by this study. In addition, the overall race times of the ten top triathletes of a nation cannot be generalized to the whole population. The nation for which triathletes compete gives no further information of their ethnicity. It is therefore possible that migration could have influenced the results of this study. Another limitation, due to the cross-sectional design of the study, is that aspects such as age [13,14], training [16,18], the influence of field tests related to training of Ironman [42], previous experience [21], and anthropometry [15-17] could not be considered.

### Practical applications

Although the US-American athletes had the fastest race times for both women and men, the number of triathletes originating from European countries such as Germany, France or Austria and Australian triathletes increased and athletes from European countries, Japan, Australia, Canada and Brazil improved race times in ‘Ironman Hawaii’. It should be considered to increase the number of slots for ‘Ironman Hawaii’ for athletes originating from Europe, Asia and South America since the performance of the annual top ten American and Australian men remained unchanged but the performance of male athletes from Brazil, Austria, Great Britain, Switzerland, Germany, Canada, Japan, New Zealand and France improved.

## Conclusion

Most participants in the ‘Ironman Hawaii’ originated from the United States of America. Male European triathletes have, in contrast to male US-American triathletes, continuously expanded their participation over the last 27 years. The fastest finishers originated for both women and men from the United States of America. The ‘Ironman Hawaii’ has been dominated in both participation and performance by female and male triathletes from the United States of America.

## Competing interests

The authors declare that they have no competing interests.

## Authors’ contributions

PD wrote the manuscript, BK collected the data, CR performed the statistical analyses, TR and RL participated in the design and coordination and helped drafting the manuscript. All authors read and approved the final manuscript.

## Pre-publication history

The pre-publication history for this paper can be accessed here:

http://www.biomedcentral.com/2052-1847/6/16/prepub
